# A New Culture Medium Rich in Phenols Used for Screening Bitter Degrading Strains of Lactic Acid Bacteria to Employ in Table Olive Production

**DOI:** 10.3390/molecules29102236

**Published:** 2024-05-10

**Authors:** Barbara Lanza, Martina Bacceli, Sara Di Marco, Nicola Simone, Giuseppina Di Loreto, Federica Flamminii, Adriano Mollica, Angelo Cichelli

**Affiliations:** 1 Council for Agricultural Research and Economics (CREA), Research Centre for Engineering and Agro-Food Processing (CREA-IT), Via Nazionale S.S. 602 km 51 + 355, 65012 Cepagatti, PE, Italy or martina.bacceli@studenti.unich.it (M.B.); sarettadimarco87@gmail.com (S.D.M.); giuseppina.diloreto@crea.gov.it (G.D.L.); 2 School of Advanced Studies, XXXVIII Cycle Ph.D. Course in Biomolecular and Pharmaceutical Sciences, University “G. d’Annunzio” of Chieti-Pescara, Via dei Vestini, 66013 Chieti, CH, Italy; 3 Council for Agricultural Research and Economics (CREA), UDG8, Via Nazionale S.S. 602 km 51 + 355, 65012 Cepagatti, PE, Italy; nicola.simone@crea.gov.it; 4 Department of Innovative Technologies in Medicine and Dentistry, University “G. d’Annunzio” of Chieti-Pescara, Via dei Vestini 31, 66013 Chieti, CH, Italy; federica.flamminii@unich.it (F.F.); angelo.cichelli@unich.it (A.C.); 5 Department of Pharmacy, University “G. d’Annunzio” of Chieti-Pescara, Via dei Vestini, 66013 Chieti, CH, Italy; a.mollica@unich.it

**Keywords:** lactic acid bacteria, multi-phase decanter, olive oil by-products, phenolic compounds, starter, table olives

## Abstract

The olive oil industry recently introduced a novel multi-phase decanter with the “Leopard DMF” series, which gives a by-product called pâté, made up of pulp and olive wastewater with a high content of phenolic substances and without pits. This study aims to create a new culture medium, the Olive Juice Broth (OJB), from DMF pâté, and apply it to select bacteria strains able to survive and degrade the bitter substances normally present in the olive fruit. Thirty-five different bacterial strains of *Lactiplantibacillus plantarum* from the CREA-IT.PE Collection of Microorganisms were tested. Seven strains characterized by ≥50% growth in OJB (B31, B137, B28, B39, B124, B130, and B51) showed a degradation of the total phenolic content of OJB ≥ 30%. From this set, *L. plantarum* B51 strain was selected as a starter for table olive production vs. spontaneous fermentation. The selected inoculant effectively reduced the debittering time compared to spontaneous fermentation. Hydroxytyrosol, derived from oleuropein and verbascoside degradation, and tyrosol, derived from ligstroside degradation, were produced faster than during spontaneous fermentation. The OJB medium is confirmed to be useful in selecting bacterial strains resistant to the complex phenolic environment of the olive fruit.

## 1. Introduction

**Figure 1:**
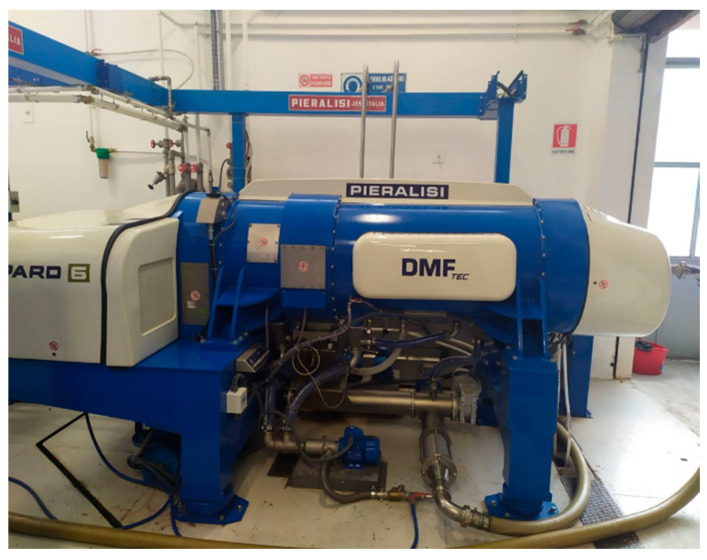
Multi-phase decanter of the Leopard DMF series.

A novelty in the olive mill extraction system is represented by the multi-phase decanters of the Leopard DMF series (Pieralisi Group S.p.A, Jesi, AN, Italy) (Figure 1), which work without the addition of processing water and with the advantage of recovering two by-products: a dehydrated pomace (dried crushed pits), which can be used as fuel for stoves and boilers, and a “pâté”, made up of pulp and olive wastewater, without traces of pits [1].

The DMF pâté represents a product with high added value for the oil mill, being able to be reused for several purposes in different fields, including agronomic (as a soil fertilizer) [2], zootechnical (as a supplement in the feeding of cattle and sheep, as it enriches milk and derivatives of polyunsaturated fatty acids and antioxidant polyphenols) [3], and energy production (as biomass for biogas plants) [4].

Pâté has a high content of phenolic compounds derived through the degradation pathway of oleuropein and ligstroside, with a prevalence of low- and medium-molecular-weight secoiridoids, such as hydroxytyrosol, tyrosol, oleacein, oleocanthal, and verbascoside [1,5,6,7,8].

Many studies have reported olive phenols’ main properties, highlighting their biological activities. Specifically, their effects on human health are anti-inflammatory, antithrombotic, anti-hyperglycemic, hepatoprotective, cardioprotective, antimicrobial, and anticancer [9,10,11,12,13,14,15,16].

Phenolic substances are known to be considered responsible for the bitter and pungent taste of extra virgin olive oil. The relationship between polyphenols and the bitterness and pungency of olive oil is complicated, and determining the thresholds of polyphenols associated with the respective sensations is difficult. Some authors have tried to associate the intensity of the bitter and pungent sensations described by the tasters of a panel with the concentrations of the respective substances, but establishing a threshold value is difficult [17,18].

Microorganisms, such as *Lactiplantibacillus plantarum*, can accelerate the debittering process by hydrolyzing the compounds that are responsible for the bitter taste of the olives [19,20,21]. The process of oleuropein and ligstroside lysis takes place in two phases: (i) the hydrolysis of the glycosidic bond by β-glucosidase, yielding the formation of oleuropein or ligstroside–aglycons; and (ii) the hydrolysis of the aglycons by esterase, with the formation of elenolic acid and hydroxytyrosol (from oleuropein–aglycon) or tyrosol (from ligstroside–aglycon) [22,23]. These latter phenols, having a non-bitter taste [17,24] and preserving their nutraceutical effects even after the processing steps, can contribute to a pleasant and healthy table olive product.

According to Paramithiotis et al. [25], lactic acid fermentation may increase the Total Phenol Content (TPC) either through the lysis of the cell wall of the plant cells and their release from the vacuole or through the β-glucosidase activity exhibited by several lactic acid bacteria strains.

Based on this scientific evidence, the scope of this research is to develop a culture medium from olive oil waste as a DMF pâté and test it for the selection of *L. plantarum* strains with high oleuropeinolytic activity. Bacterial strains will then be classified in order of their ability to survive and degrade the bitter substances normally present in the olive fruit, and they could subsequently be used to obtain a quicker and/or more efficient debittering of olives intended to produce table olives. Finally, this culture medium could be standardized and become a reference as a selective medium for microorganisms resistant to phenols and/or capable of degrading complex phenolic substances.

## 2. Results

Our results include data on DMF pâté’s chemical–physical characterization (Table 1), the use of Olive Juice Broth (OJB) by LABs, and the behavior of the better-performing strain applied to a fermentation trial.

Due to the content of organic acids present in olives (mainly malic and citric acid), the Dritta cv. pâté shows an acidic pH (about 4.5) and a high acidity (0.716). These characteristics could prevent Gram-negative microorganisms’ growth and promote lactic acid bacteria development.

The water activity of food expresses the amount of bioavailable water for microorganisms, and it is used by the Food and Drug Administration to establish thresholds from which foods are subject to mold attacks. Values can be included between 0 and 1, which represents a high possibility of microorganism development. The high value obtained for our pâté (0.9883) is surely due to the high intrinsic moisture (M) of the product (80.89%). However, this moisture value was lower than that found in other pâtés previously analyzed and derived from different cultivars [1], in which water was presumably added to the process.

The high values of ashes, dry matter (DM), organic matter (OM), and organic carbon (OC) (Table 1) indicate that the pâté has a high concentration of organic substances, such as sugars, pectins, nitrogenous substances, polyalcohols, and polyacids. These characteristics may represent growth factors for microorganisms and therefore be important in the production of a culture medium because they would limit the addition of extra components.

A high oil content (>7%) was observed, which may be because the cultivar Dritta is very rich in oil, and so there could be a higher residue after the oil extraction process [26].

Also, the color was evaluated as an important attribute to monitor the oxidation degree of a vegetal matrix. The lightness value (L*) of our sample indicates a dark-brownish color tendency. Positive values for a* and b* suggest redness and yellowness, and CHROMA (chromaticity) reveals a matte color.

In Table 2 are displayed results obtained about fatty acid composition (in percentage), with the mean and the standard deviation of the oil extracted from the pâté using a Soxhlet system compared with the mean fatty acid composition of monovarietal extra virgin olive oils (MEVOO) regarding the cv. Dritta. As shown in Table 2, the mean data of this study are very similar to those published for this cultivar [26]. The oily fraction of the analyzed pâtés contains optimal percentages of ω-3, ω-6, and ω-9 and does not contain trans-fatty acids. In particular, the fatty acid composition reveals average characteristics close to high-quality olive oil [27], with MUFA higher than 70% and a high ratio of MUFA/SFA.

Table 3 shows all phenolic compounds detected in the olive juice before and after UV sterilization. UV irradiation is a widespread method to sterilize both solid and liquid foods, being included in non-thermal technologies suitable for minimizing the loss of nutrients and sensory properties with low energy demand [28]. This method allowed us to obtain sterilized juice as a starting point to make the new medium. Its observation revealed an increase in many phenolic compounds, such as hydroxytyrosol and tyrosol, even if the final total content was reduced.

The decrease in Total Phenol Content (TPC), although minimal, was expected because of the oxidizing action of the light [29], but this step could not be skipped, due to the need to sterilize the substrate to be inoculated.

As specified in the Introduction, this study aimed to create a new culture medium, the Olive Juice Broth (OJB), from DMF pâté, and apply it to select bacteria strains able to survive and degrade the bitter substances normally present in the olive fruit.

Thirty-five different bacterial strains of *Lactiplantibacillus plantarum* from the CREA-IT.PE Collection of Microorganisms tested their abilities.

As shown in Table 4, the results are encouraging for several strains. Seven of them show a survival percentage higher than 50% and a quite high percentage of TPC degradation. In column 2 is shown the survival distribution of the 35 L plantarum strains in OJB with respect to the control in MRS broth. In column 3 is shown the total phenolic content residual of the inoculated samples compared to the initial phenolic content. All seven strains characterized by ≥50% growth in OJB (B31, B137, B28, B39, B124, B130, and B51) showed a degradation of the total phenolic content of OJB ≥ 25%.

**Figure 2:**
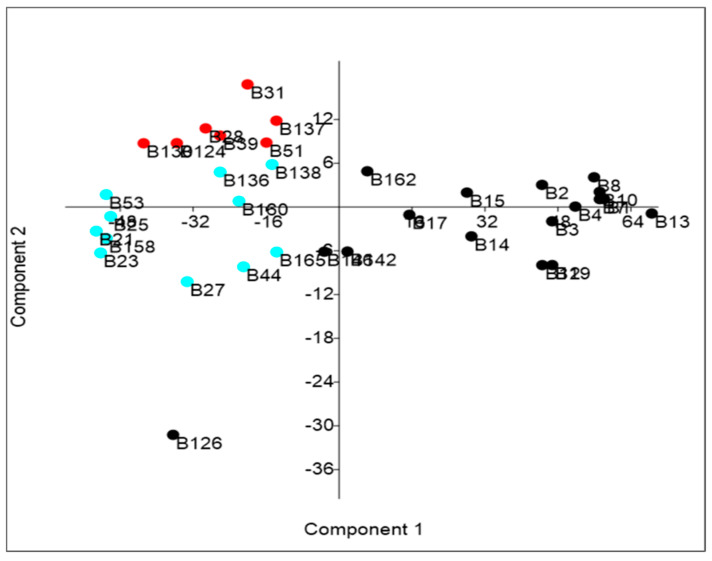
Bi-plot obtained through PCA.

The PCA bi-plot reported in Figure 2 shows the differences between strains. Table 5 shows the variance explained by the principal components (variance > 95% is explained by PC1).

As shown in Table 4 and Figure 2, in addition to the seven promising strains (in red in Figure 2), there are another eleven strains (in light blue in Figure 2; B138, B136, B53, B160, B25, B21, B158, B23, B165, B44, and B27) that could be considered interesting as they show both a very high % of survival and % of degradation. Strain B126 shows a high % of TPC degradation but an extremely low % of growth, highlighting its poor tolerance to degradation products.

Then, two different fermentations were evaluated: A, spontaneous fermentation; B, fermentation with an inoculum (*L. plantarum* B51). The choice of this microorganism depended on survival and degradation tests and previous technological/probiotic characterization [1,30,31].

We followed the entire fermentation process on Itrana cv. olives to test the degradation of bitter phenolic compounds, such as oleuropein, ligstroside, and verbascoside, by quantifying the production of their non-bitter derivatives, such as hydroxytyrosol and tyrosol. Throughout the fermentation process, titratable acidity and pH values were monitored, and microbiological analyses were performed to check the existing microflora.

**Figure 3:**
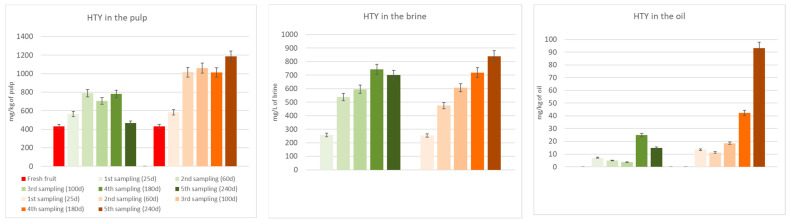
Hydroxytyrosol (HTY) changes in the pulp, brine, and oil fraction during spontaneous (in green) and inoculated (in orange) fermentations.

**Figure 4:**
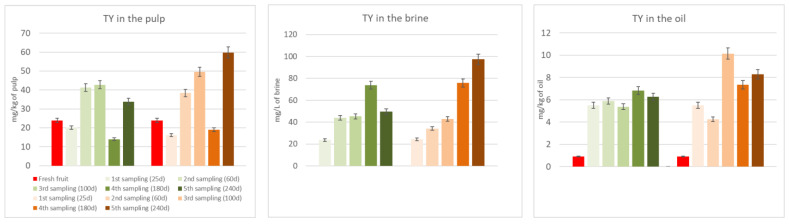
Tyrosol (TY) changes in the pulp, brine, and oil fraction during spontaneous (in green) and inoculated (in orange) fermentations.

Figure 3 and Figure 4 show the biophenols’ (hydroxytyrosol and tyrosol) changes occurring in the pulp, oil, and brine portions during both spontaneous and inoculated fermentations. As it can be seen in the figures, hydroxytyrosol and tyrosol contents increased in all fractions compared to fresh fruit, especially in the inoculated olives, thanks to the hydrolysis of the initial glucosides. Their high production is notably clear in the two last samplings, after 180 and 240 days from the beginning of the fermentation process.

## 3. Discussion

The Dritta DMF pâté is characterized by the following:

- A high content of organic matter;
- A high oil content (>7%) whose fatty acid composition is very close to that of extra virgin olive oils that are extraordinarily rich in oleic acid and with a good percentage of linoleic acid;
- A high content of phenolic compounds, which are mainly represented by secoiridoids and verbascoside.

Based on these characteristics, we used this substrate to prepare a new culture medium (Olive Juice Broth—OJB) to select microorganisms, principally lactic acid bacteria, capable of both growing in the presence of high phenol concentrations and degrading the phenols responsible for the bitter taste of the fruit into simple, non-bitter phenols. Thanks to its high concentration of organic substances, we added as growth factors only 2% of glucose and 0.4% of yeast extract. An important aspect is also the recent trend of identifying new sources of nutrients, particularly those free of animal components, which could be used to prepare new culture media based on sustainable sources, with a zero or negative carbon footprint and with no animal origin. The new culture medium OJB prepared with DMF olive waste (pâté) could be a suitable alternative to conventional media, such as MRS, a medium based on animal components, to select lactic acid bacteria. In addition to phenol compounds, protein and sugar content are naturally available in DMF pâté as a source of nitrogen and carbohydrates, to which we have added yeast extract and glucose as growth factors.

To carry out this trial (lab-scale olive fermentation), olives at the green ripening stage were chosen because of their abundance of bitter substances. Characterizing in a proper way the raw material used to obtain OJB in our tests is important because of the nature of the product. The chemical and physical parameters of the olive fruit are due to many different variables, such as geographical position, sun period, mean temperature, temperature slope, rain/irrigation, soil composition, different fertilization, supply of organic carbon to the soil, biotic and abiotic stresses, and many more. All of these variables affect the chemical and physical composition of the fruit, and they can vary year by year, so we found of primary importance a deep characterization, especially to repeat and/or reproduce the experiments with a similar product and to standardize OJB production.

Table 3 shows the TPC of the DMF pâté before and after the UV sterilization, which is an indispensable pre-inoculum step. The lowering of the TPC is probably due to the oxidizing action of the light. In any case, this phase resulted in a slight rise of hydroxytyrosol and tyrosol, probably thanks to the hydrolysis of oleuropein and ligstroside derivatives, which, indeed, decreased. The results obtained pushed us to focus our attention on seven promising strains to use as starters in table olive processing during the debittering phase. Two different fermentations were evaluated: A, spontaneous fermentation; B, fermentation with an inoculum (*Lactiplantibacillus plantarum* B51). Due to previous research showing its probiotic activity [1,30,31] and due to its position in survival percentage and TPC degradation (Table 4), our choice for a specific fermentation test fell on the B51 strain. Table 4 shows the performance of this strain in terms of its ability to survive in the OJB medium and its debittering activity. The selected inoculant seems to reduce the debittering time; hydroxytyrosol, the major non-bitter compound derived from oleuropein and verbascoside degradation, and tyrosol, the major non-bitter compound derived from ligstroside degradation, were produced faster than through spontaneous fermentation. This phenomenon can easily be observed in Figure 3 and Figure 4.

Moreover, in Figure 3 and Figure 4, we can observe the quite constant rise of hydroxytyrosol and tyrosol in the brine, pulp, and oil of the inoculated samples. This trend is not observed in the spontaneously fermented samples.

## 4. Materials and Methods

### 4.1. Olive Pâté Sampling

**Figure 5:**
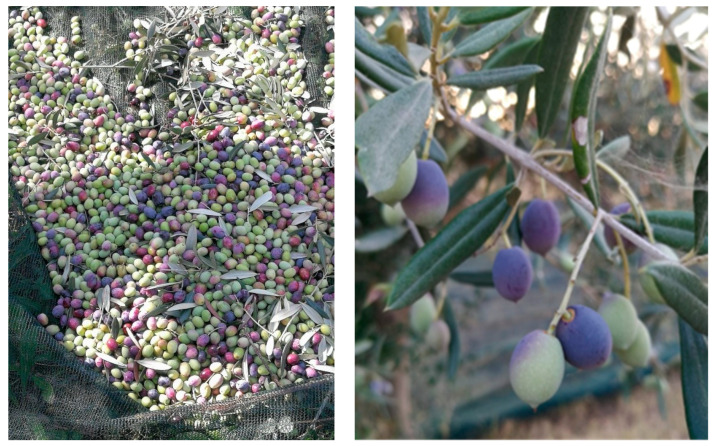
Olives from *Olea europaea* L. “Dritta” cv.

Olives from *Olea europaea* L. “Dritta” cv (Figure 5) were collected during November in the middle area of Italy (Abruzzo Region) and processed through a modern multi-phase centrifugal plant (Leopard DMF 6 Series, Pieralisi Group S.p.A., Jesi, AN, Italy) located in the oil mill SCAL (Loreto Aprutino, PE, Italy). The very-wet pomace (pâté) samples were placed in thermal bags and quickly brought to the laboratory, where they were immediately frozen and then stored at −20 °C until use.

### 4.2. Characterization of Fresh DMF Pâté

#### 4.2.1. pH, Titratable Acidity, Moisture, Ash, and Oil

The pH of the pâté was measured with an Istek pH Meter 730P model (Istek, Inc., Seoul, Republic of Korea).

The titratable acidity was determined by titrating 20 g of pâté with NaOH 0.1 N solution using phenolphthalein as an indicator 1% (*w*/*v*) solution in ethanol. The results are expressed in grams of citric acid per 100 g of fresh pâté.

The moisture (M) was determined through a drying process. Homogenized samples (about 20 g) were placed and weighed in a Pyrex crucible, previously dried at 105 °C up to a constant weight. The samples were put in an oven at 105 °C for at least 24 h until constant weight was reached (±0.005 g). The moisture content was calculated as the difference in weight and expressed in % up to the second decimal. To determine the ash content, homogenized samples (about 5 g) were placed and weighed in a porcelain crucible, previously dried at 105 °C up to a constant weight. Then, they were heated to 550 °C in a thermostatically controlled muffle furnace for at least 24 h and until reaching a whitish brown color. Dry matter (DM) was calculated using the formula DM = 100-M. Organic matter (OM) was calculated through the formula OM = 100-M-ash. Organic carbon (OC) was calculated considering 58% of OM.

The oil content was determined using a Soxhlet extractor. The oil was extracted from the dried olive pulp used for moisture determination through repeated washing (percolation) with petroleum ether 40–60 under reflux in a special glassware. The ether was removed through evaporation, and the residual oil was weighed.

#### 4.2.2. Fatty Acid Composition

The fatty acid composition of the oil was determined according to the method described in European Union Commission Regulation EEC/2568/91 and its subsequent modifications (Annex X.B). The procedure employs a gas chromatography system (HRGC Mega 2 series 8560; Carlo Erba, Milan, Italy) equipped with an SPTM-2380 (Supelco, Bellefonte, PA, USA) fused silica capillary column (60 m × 0.32 mm ID × 0.2 µm film thickness). The oven temperature program was from 70 °C to 165 °C at 20 °C min^−1^ and held at 165 °C for 23 min; then from 165 °C to 200 °C at 1.5 °C min^−1^ and held at 200 °C for 5 min; and then from 200 °C to 220 at 2 °C min^−1^ and held at 220 °C for 5 min. The detector temperature was 230 °C. Hydrogen was used as the carrier gas at a column head pressure of 60 kPa. The samples (0.4 µL) were applied through on-column injection.

#### 4.2.3. Activity Water (a_w_)

a_w_ was determined using the AquaLab Pre Water Activity Meter (METER Group, Pullman, WA, USA). An initial calibration with two standard solutions (a_w_ 0.760 and a_w_ 0.920) was carried out. The analysis was performed by taking triplicate pâté samples at room temperature.

#### 4.2.4. Color

The color of the pâté was measured using a Colour-view spectrophotometer (Konica Minolta Optics, 2970 Ishikawa-machi, Hachioji, Tokyo, Japan; Model CM-2600D). The color was expressed in terms of CIE (Commission Internationale de l’Eclairage) L* (lightness), a* (redness/greenness), b* (yellowness/blueness), and their derivative Chroma (C = √(a*^2^ + b*^2^)). L* has values ranging from 0 (dark) to +100 (light), a* is from −120 (red) to +120 (green), and b* is from +120 (yellow) to −120 (blue).

### 4.3. Preparation of the Olive Juice Broth (OJB)

**Figure 6:**
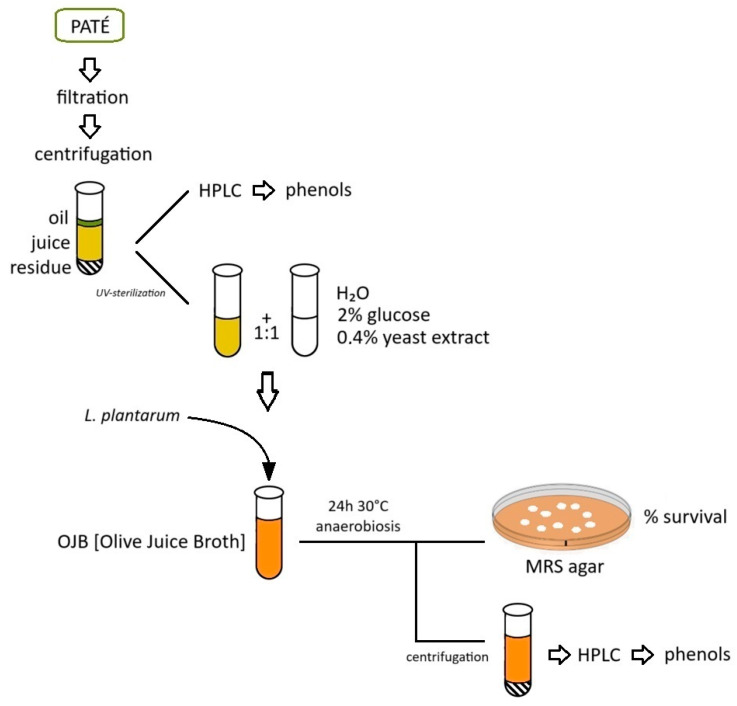
Preparation of the Olive Juice Broth (OJB) and tests on microorganisms.

The preparation of the Olive Juice Broth (OJB) is shown in Figure 6. After refrigerated transport, the samples of the pâté collected at the decanter recovery were filtered with a vacuum pump through decreasing pore-size sieves (smallest pore size 0.45 µm) and then centrifuged twice (3756× *g*, 15 min, 4 °C). Once the oily part on the surface was removed, the supernatant or “juice” was recovered. An aliquot of the juice was treated for subsequent injection into HPLC for the determination of the initial phenolic content; the remaining juice was sterilized under UV for 1 h and then added, in a 1:1 ratio, to an autoclave-sterilized solution of demineralized water containing 2% glucose and 0.4% yeast extract, thus obtaining an enriched culture medium (OJB—Olive Juice Broth) on which to test oleuropeinolytic microorganisms. The test tubes containing OJB and microorganisms were incubated for 24 h at 30 °C in an anaerobic atmosphere against a growth test in MRS broth. The percentage of survival in OJB was assessed through growth in MRS agar. Aliquots of juice, before and after UV sterilization, and degraded OJB were processed in HPLC for the determination of the initial and residual phenolic content.

### 4.4. Test of Microorganisms’ Survival in OJB

An aliquot of OJB was used to be injected into HPLC, and the remainder was divided into 35 sterile tubes, each inoculated with a different bacterial strain of *L. plantarum* from the CREA-IT.PE Collection of Microorganisms. The tubes were incubated for 24 h at 30 °C against growth in MRS broth. The growth of the strains (coming from OJB and MRS broth) was evaluated through the growth in MRS agar, and the results are expressed as colony-forming units (CFU) per mL. Then, the survival percentage was calculated by applying the following formula:

% survival = (log CFU/mL _OJB_)/(log CFU/mL _MRS_) × 100

### 4.5. Test of Microorganism’s Phenolic Degradation in OJB

The contents of each tube were centrifuged (3756× *g*, 15 min, 4 °C) to precipitate the bacterial suspension. The supernatant was processed to be injected into HPLC to obtain the residual phenolic content.

### 4.6. Spontaneous and Controlled Lab-Scale Fermentation Tests

The fruits of *Olea europaea* L. Itrana cv. were hand-harvested at their mature–green stage of ripening (1.9 Jaen Index) during December. The olives were divided into four lots of 2.7 kg of olives (about 700 drupes) and placed in glass jars, then submerged in 8% brine prepared with 80 g/L of sterilized Trapani PGI sea salt, following the Greek-style method. Two different fermentations were evaluated: A, spontaneous fermentation; B, fermentation with an inoculum (*Lactiplantibacillus plantarum* B51). The choice of this microorganism depended on tests described in 4.4 and 4.5 and the technological/probiotic characterization described in previous papers [1,30,31]. The bacterial precultures of *L. plantarum B1* (5 mL) were transferred into flasks of 100 mL of MRS broth and incubated for 24 h at 30 °C to obtain the inoculum biomass; the cells were centrifuged at 3756× *g* for 15 min at 15 °C, and the pellet was re-suspended in 100 mL of sterilized brine (80 g/L of Trapani PGI sea salt). The bacterial precultures of *L. plantarum* B51 (2.5 mL) were transferred into flasks of 50 mL of MRS broth and incubated for 24 h at 30 °C to obtain the inoculum biomass; the cells were centrifuged at 3756× *g* for 15 min at 15 °C, and each pellet was re-suspended in 100 mL of sterilized brine (80 g/L of Trapani PGI sea salt). After 1 week from the immersion of the olives in the brine, the bacterial culture (inoculum level: 6 log/mL) was distributed into the glass jars and then incubated at room temperature. The sampling was performed during the whole fermentation process after 25, 60, 100, 180, and 240 days. We determined the end of fermentation at the 5th sampling, based on the chemical–physical–microbiological parameters analyzed. The study was conducted in duplicate.

### 4.7. Monitoring of Fermentation Process

Throughout the fermentation process, titratable acidity and pH values were monitored, and microbiological analyses were performed to check the existing microflora.

### 4.8. Oil Extraction and Pulp Recovery from Fresh Olive Fruits

Each sample was made up of 30 olive fruits, manually de-pitted, and triturated with a grinder. The olive paste was warmed up and mixed every 5 min in a water bath at 28 °C for 30 min, and then it was centrifuged at 3756× *g* for 30 min at 10 °C in a refrigerated centrifuge. The resulting supernatant oil was collected with a Pasteur pipette, filtered in the presence of anhydrous sodium sulphate to make it moisture-free, stored in 10 mL Falcon^®^plastic tubes (Thermo Fisher Scientific Inc., Waltham, MA, USA) wrapped with aluminium foil to keep them in the dark, and finally kept at 4 °C until the analysis. This procedure simulates the extraction of olive oil in olive mills (i.e., crushing, malaxation, and centrifugation), and it was used to prevent changes in the oil quality as best as possible.

### 4.9. Biophenols

The extraction of the phenolic substances in the pâté juice was performed according to the International Olive Council method COI/T.20/Doc No 29/2009, adapted as explained below. First, 1 mL of juice was transferred to a test tube with 1 mL of internal standard (syringic acid 0.015 mg/mL in methanol/water 80/20 *v*/*v*) and vortexed for 30 s. Then, 5 mL of methanol/water 80/20 *v*/*v* was added to the mixture, vortexed for 1 min, sonicated in an ultrasonic bath for 15 min, and centrifuged at 3756× *g* for 5 min at room temperature. A 1 mL aliquot of the resulting upper phase was filtered in PVDF 0.45 µm (Merck, Darmstadt, Germany) to be analyzed. The HPLC analysis of the phenolic extracts was carried out using an LC 200 high-resolution liquid chromatograph, equipped with a Series 200 UV/Vis detector (Perkin Elmer, Waltham, MA, USA), a 7725 Rheodyne injector, a 20 µL sample loop, and a Totalchrom workstation (Perkin Elmer, Waltham, MA, USA) for data acquisition. Separation on a Spherisorb ODS2 column (250 × 4.6 mm I.D., 5 µm; Waters, Milford, MA, USA) was performed at 25 °C under a constant flow rate of 1 mL/min and the following ternary gradient program (in %): t = 0 min, A (water 0.2% H_3_PO_4_ (*v*/*v*))/B (methanol)/C (acetonitrile) = 96/2/2 *v*/*v*/*v*; t = 40 min, A/B/C = 50/25/25 *v*/*v*/*v*; t = 45 min, A/B/C = 40/30/30 *v*/*v*/*v*; t = 60 min, A/B/C = 0/50/50 *v*/*v*/*v*; t = 70 min, A/B/C = 0/50/50 *v*/*v*/*v*; t = 72 min, A/B/C = 96/2/2 *v*/*v*/*v*; t = 82 min, A/B/C = 96/2/2 *v*/*v*/*v*. Eluted compounds were detected at 280 nm. First, 20 µL of the external calibration standard solution was injected to calculate the values of the response factors for 0.030 mg/mL of tyrosol and 0.015 mg/mL of syringic acid. Finally, 20 µL of the sample solution was injected into the HPLC system. Quantification of the phenolic compounds was performed according to the concentration of the internal standard and based on the response factor.

Regarding the extraction of the phenolic fraction content of the pulp, the oil, and the brine of the lab-scale fermentation samples, the following procedure was adopted.

To prepare the phenolic extract of olive pulp, 0.5 g of homogenized pulp (weight considered to the fourth decimal place) was transferred into a test tube with 1 µL of internal standard (syringic acid 0.015 mg/mL in methanol/water 80/20 *v*/*v*) and vortexed for 30 s. The mixture was added with 5 mL of methanol/water 80/20 *v*/*v*, vortexed for 1 min, sonicated in an ultrasonic bath for 15 min, and centrifuged at 3756× *g* for 5 min at room temperature, and 1 mL of the supernatant was filtered in PVDF 0.45 µm (Merck, Darmstadt, Germany) to be analyzed.

To prepare the phenolic olive oil extracts, 2.500 g oil was added to 500 µL of internal standard solution (syringic acid 0.015 mg/mL in methanol/water 80/20 *v*/*v*). After the removal of the methanol under reduced pressure at a temperature of <35 °C, the sample was dissolved in 6 mL of hexane and loaded onto an SPE column (Discovery DSCDIOL 500 mg, 6 mL; Supelco, Bellefonte, PA, USA) previously conditioned with 6 mL of methanol and 6 mL of hexane. The sample was then subjected to washing twice with 3 mL of hexane and finally with 4 mL of hexane/ethyl acetate 90/10 and then extracted with 10 mL of methanol. After the removal of the methanol with a rotary evaporator at a temperature of <35 °C, the dry residue was taken up with 1 mL of methanol/water 50/50 *v*/*v*, and an aliquot of 20 µL was injected into the HPLC system after filtration on a 0.45 µm PVDF membrane.

Simultaneously with the analysis of the olive extracts, the phenol composition of the fermentation brine was monitored. A volume of 1 mL of brine was transferred to a 5 mL volumetric flask, made up of volume with the solution of methanol/water 80/20 (*v*/*v*), and filtered through a 0.45 µm PVDF membrane. Finally, 20 µL of the sample solution was directly injected into the HPLC system.

All of the analyses were carried out in duplicate for each sample.

### 4.10. Statistics

Data derived through survival and degradation tests were processed through principal component analysis (PCA), carried out in the Past PAleontological STatistics software (Version 2.12, Øyvind Hammer, Natural History Museum, University of Oslo, Norway).

## 5. Conclusions

The results obtained show that seven LAB strains (B31, B137, B28, B39, B124, B130, and B51) survived by more than 50% in an OJB medium and led to a significant decrease in the total phenolic content. Other strains degrade TPC with a higher percentage, yet they have not been considered due to low survivability (lower than 50%). Between those seven strains, the *L. plantarum* B51 strain was our elected choice due to its intrinsic probiotic activity shown in previous research, coupled with the ability to survive in OJB and the ability to achieve a reduced debittering time when inoculated in olive fermentation, while adding probiotic characteristics to the final product. As expected, it was demonstrated to be a concrete advantage compared to spontaneous fermentation.

The OJB medium developed from DMF pâté can be confirmed as a cheap and useful way to grow and select bacterial strains resistant to the complex phenolic environment of the olive fruit. This could also be useful in selecting strains for the biological degradation of the olive mill’s wastewater and other liquid or solid wastes rich in phenolic substances. Further and deeper studies involving single and multiple inocula of the best surviving strains should be repeated on table olive industrial processes based on natural fermentation due to the increased efficiency in degrading the bitter substances, resulting in brines with a lower TPC and, consequently, lower COD and BOD_5_, resulting in a more environmentally friendly waste.

## Figures and Tables

**Table 1:** Chemico-physical characteristics of fresh DMF pâté. The values are expressed as mean ± standard deviation.

Parameter	Value
pH	4.56 ± 0.03
Titratable acidity (citric acid g/100 g fresh pâté)	0.716 ± 0.015
Activity water (a_w_)	0.9883 ± 0.0025
Moisture (%)	80.89 ± 0.03
Ash (g/100 g fresh pâté)	1.10 ± 0.05
DM (g/100 g fresh pâté)	19.11
OM (g/100 g fresh pâté)	18.01
OC (g/100 g fresh pâté)	10.45
Oil content (g/100 g fresh pâté)	7.30 ± 0.10
Color	
L*	29.73 ± 4.94
a*	9.74 ± 2.28
b*	26.55 ± 9.16
CHROMA	28.29 ± 9.38

**Table 2:** Fatty acid composition of oil fraction of DMF pâté. The values are expressed as mean ± standard deviation. MEVOO, monovarietal extra virgin olive oil.

Figure 1	DMF Pâté	MEVOO Dritta cv. ^1^	MEVOO Dritta cv. ^2^
C14:0 (Myristic acid)	0.06 ± 0.01		
C16:0 (Palmitic acid)	12.98 ± 0.07	13.75 ± 1.36	13.11 ± 0.35
C16:1 (Palmitoleic acid)	0.89 ± 0.05	0.96 ± 0.20	1.26 ± 0.07
C17:0 (Heptadecanoic acid)	0.05 ± 0.01	0.07 ± 0.02	
C17:1 (Heptadecenoic acid)	0.07 ± 0.01	0.08 ± 0.02	
C18:0 (Stearic acid)	2.98 ± 0.07	2.65 ± 0.46	1.85 ± 0.01
C18:1 ω9 (Oleic acid)	72.18 ± 0.13	72.83 ± 2.58	74.77 ± 0.74
C18:2 ω6 (Linoleic acid)	9.33 ± 0.05	8.23 ± 1.35	8.02 ± 0.12
C20:0 (Arachidic acid)	0.43 ± 0.00	0.43 ± 0.07	0.18 ± 0.02
C18:3 ω3 (α-Linolenic acid)	0.65 ± 0.01	0.67 ± 0.08	0.71 ± 0.02
C20:1 (Eicosenoic acid)	0.22 ± 0.00	0.25 ± 0.07	
C22:0 (Behenic acid)	0.13 ± 0.00		
C24:0 (Lignoceric acid)	0.03 ± 0.01		
SFA	16.66	16.90	15.14
MUFA	73.36	74.12	76.03
PUFA	9.98	8.90	8.73
Oleic acid/Linoleic acid	7.74	8.85	9.32
Oleic acid/Palmitic acid	5.56	5.30	5.7
Linoleic acid/Palmitic acid	0.72	0.60	0.61
MUFA/SFA	4.40	4.39	5.02
PUFA+MUFA/SFA	5.00	4.91	5.59
PUFA/SFA	0.60	0.53	0.57
omega6/omega3	14.35	12.28	11.29

^1^ Italian National Database of Monovarietal Extra Virgin Olive Oils (https://www.olimonovarietali.it/en/database/detail/?id=DRITTA&regione=ABRUZZO (accessed on 24 January 2024). ^2^ Flamminii et al. [26].

**Table 3:** Single phenol profile of olive juice pre- and post-UV sterilization. The values are expressed as mg/kg mean ± standard deviation.

Phenolic Compound (mg/kg)	Olive Juice Pre-UV	Olive Juice Post-UV
3,4-DHPEA (Hydroxytyrosol)	127.5 ± 22.2	138.0 ± 19.5
*p*-HPEA (Tyrosol)	14.6 ± 2.8	15.6 ± 2.5
*p*-Hydroxybenzoic acid	61.5 ± 11.2	63.4 ± 6.3
Vanillic acid	48.1 ± 10.7	34.4 ± 3.5
Caffeic acid	17.8 ± 5.8	17.4 ± 4.4
Vanillin	10.3 ± 3.8	17.4 ± 2.5
*p*-Coumaric acid	12.6 ± 4.9	25.2 ± 7.0
Hydroxytyrosyl acetate	11.9 ± 4.3	10.6 ± 1.4
Ferulic acid	18.7 ± 5.8	65.8 ± 5.4
Verbascoside	47.8 ± 5.2	47.5 ± 4.9
*o*-Coumaric acid	60.3 ± 7.0	37.8 ± 6.8
3,4-DHPEA-EDA (Oleacein)	29.6 ± 8.5	38.6 ± 6.6
Oleuropein	17.8 ± 1.7	9.1 ± 3.1
3,4-DHPEA-EA (Oleuropein aglycone)	12.1 ± 1.2	26.2 ± 2.5
Tyrosyl acetate	15.1 ± 1.3	37.4 ± 2.2
Rutin	49.5 ± 7.5	48.7 ± 12.1
*p*-HPEA-EDA (Oleocanthal)	25.0 ± 9.9	39.4 ± 5.2
Pinoresinol, 1-Acetoxypinoresinol	4.8 ± 1.3	4.0 ± 0.2
Cinnamic acid	0.9 ± 0.1	0.8 ± 0.1
*p*-HPEA-EA (Ligstroside aglycone)	2.8 ± 0.9	4.4 ± 0.9
Luteolin	5.5 ± 1.7	5.4 ± 0.5
3,4-DHPEA,-EA,H	8.5 ± 1.6	6.2 ± 0.7
Apigenin	0.0	0.0
7-*O*-Methyl-luteolin	0.0	0.0
*p*-HPEA,-EA,H	0.9 ± 0.2	2.0 ± 0.1
Total Phenol Content (TPC)	554	349

**Table 4:** Survival and TPC degradation percentage for all tested microorganisms.

	% Survival	TPC Residual (mg/kg)	% TPC Degradation
B31	59	256	27
B137	54	262	25
B28	53	247	29
B39	52	250	28
B124	51	241	31
B130	51	234	33
B51	51	260	26
B138	48	261	25
B162	47	281	19
B136	47	250	28
B8	46	329	6
B2	45	318	9
B15	44	302	13
B10	44	330	5
B53	44	226	35
B1	43	331	5
B7	43	330	5
B160	43	254	27
B4	42	325	7
B25	41	227	35
B17	41	290	17
B13	41	341	2
B3	40	320	8
B21	39	224	36
B158	38	226	35
B14	38	303	13
B146	36	272	22
B23	36	225	36
B142	36	277	21
B165	36	262	25
B44	34	255	27
B19	34	320	8
B12	34	318	9
B27	32	243	30
B126	11	240	31

**Table 5:** Eigenvalue and percentage variance contributions by PCs of the data set.

PC	Eigenvalue	% Variance	Cumulative Variance
1	1585.570	95.620	95.620
2	72.558	4.370	99.990
3	0.068	0.010	100.000

## Data Availability

The original contributions presented in the study are included in the article, further inquiries can be directed to the corresponding authors.
